# An Application of Self-Organizing Map for Multirobot Multigoal Path Planning with Minmax Objective

**DOI:** 10.1155/2016/2720630

**Published:** 2016-06-02

**Authors:** Jan Faigl

**Affiliations:** Department of Computer Science, Faculty of Electrical Engineering, Czech Technical University in Prague, Technická 2, 166 27 Prague 6, Czech Republic

## Abstract

In this paper, Self-Organizing Map (SOM) for the Multiple Traveling Salesman Problem (MTSP) with minmax objective is applied to the robotic problem of multigoal path planning in the polygonal domain. The main difficulty of such SOM deployment is determination of collision-free paths among obstacles that is required to evaluate the neuron-city distances in the winner selection phase of unsupervised learning. Moreover, a collision-free path is also needed in the adaptation phase, where neurons are adapted towards the presented input signal (city) to the network. Simple approximations of the shortest path are utilized to address this issue and solve the robotic MTSP by SOM. Suitability of the proposed approximations is verified in the context of cooperative inspection, where cities represent sensing locations that guarantee to “see” the whole robots' workspace. The inspection task formulated as the MTSP-Minmax is solved by the proposed SOM approach and compared with the combinatorial heuristic GENIUS. The results indicate that the proposed approach provides competitive results to GENIUS and support applicability of SOM for robotic multigoal path planning with a group of cooperating mobile robots. The proposed combination of approximate shortest paths with unsupervised learning opens further applications of SOM in the field of robotic planning.

## 1. Introduction

Self-Organizing Map (SOM) is an unsupervised neural network proposed by Kohonen in 1982 as a technique to map a high-dimensional input space into a lower dimensional (usually 2D) output space. Although SOM has been originally proposed for data visualization, it has been applied to many other problems including a solution of the Traveling Salesman Problem (TSP) [1]. The TSP stands to find a closed shortest tour to visit a given set of cities (locations) such that each city is visited exactly once and the tour returns to the starting city. It is known that the TSP is NP-hard and it is a well studied problem in operational research [2], where efficient heuristics have been proposed [3, 4].

On the other hand, the earliest application of SOM to the TSP was proposed independently by Angéniol et al. [5] and Fort [6] in 1988. Since that, several approaches have been developed to improve performance of the unsupervised learning of SOM for the TSP, for example, by a combination with *λ*-opt heuristic [7], using inhibition mechanism [8], considering geometric properties of the associated solution [9], and so forth; see extensive overviews in [10–12]. However, most of the approaches consider the Euclidean variant of the TSP in which cities are locations in a plane. Although few works on SOM for other routing problems have been published [13–16], non-Euclidean TSP is relatively unnoticed by the research community. It is probably because of the main difficulty of SOM for the non-Euclidean TSP that is a determination of the best matching neuron to the input signal presented to the network. It can be nontrivial to evaluate a suitable distance function and thus it can decrease performance of any algorithm based on elastic net principles [13].

For the unsupervised learning of SOM for the TSP, the best matching neuron is determined as the distance between the neuron weights and cities, which can be easily computed as the Euclidean distance. In robotic planning, the problem is to find a shortest path to visit a given set of cities and such a distance corresponds to the length of the shortest path among obstacles, which is more computationally demanding than computation of the Euclidean distance. The requirement for collision-free paths connecting the particular cities in the tour is the main reason why the problem is called the multigoal path planning (MTP) rather than the TSP to emphasize this difficulty [17]. Therefore, we aim to extend existing SOM approaches for the TSP to address more challenging MTP problems.

A simple and fast approximation of the shortest path in the polygonal domain *𝒲* has been proposed in [18] that enables to deploy the SOM for the TSP [8] to the robotic MTP. In this paper, the approximation is further developed to address the multirobot variant of the MTP, where *k* shortest paths (for *k* robots) among obstacles are requested to visit the given set of locations in the polygonal domain. The addressed problem is considered as a variant of the Multiple Traveling Salesman Problem (MTSP) with minmax objective [19] in which we aim to minimize the longest tour. This variant has a suitable objective function for motivational inspection planning or search and rescue scenarios, where it is desired to search the given environment as quickly as possible, and the total mission time corresponds to the length of the longest path a robot has to travel [20].

This presented work reports on an extension of the SOM for the MTSP with minmax objective proposed in [16] to a more general approach for the multirobot multigoal path planning problem to visit a given set of locations in the polygonal domain *𝒲*. The proposed approach is based on our previous work on approximation of the shortest path in *𝒲* for SOM-based solution of routing problems [18, 20, 21]. Therefore, we focus on an evaluation of the proposed extensions and an alternative inexpensive procedure for the competitive rule for the SOM-based MTSP-Minmax. The performance of the proposed approach is compared with a combinatorial heuristic algorithm for the MTSP-Minmax called GENIUS [22]. The presented results indicate that the proposed extensions make SOM competitive with the combinatorial approach from the solution quality and required computational time points of view. Furthermore, solutions found by SOM provide interesting features in relation to the robotic motivational problem, where SOM tends to provide mutually noncrossing tours for the robots.

The rest of the paper is organized as follows. An overview of the related work is presented in the next section. The problem statement, used notation, and terminology are introduced in Section 3. A detailed description of the selected reference combinatorial algorithm [22], the considered SOM for the MTSP [16], and utilized approximation of the shortest path in *𝒲* are presented in Section 4 to provide a better understanding of the proposed extensions and the evaluated algorithms' variants. The proposed extensions of the SOM for the MTSP [16] to address the multirobot MTP problem are presented in Section 5. Evaluation results and comparisons of the algorithms in several problems motivated by the inspection planning are reported in Section 6. Conclusion and remarks about further work are discussed in Section 7.

## 2. Related Work

Various problems can be formulated as the TSP or MTSP, but in our case, the considered problem is motivated by path planning problems in inspection and search missions, where single or a group of mobile robots is requested to visit a given set of locations as quickly as possible. The problem is called the multigoal path planning (MTP) problem [17, 23] in robotics, and the additional problem to the standard formulation of the MTSP is the necessity to consider paths among obstacles to avoid possible collision of the robots with obstacles in the workspace.

For a simple case, when paths between two locations are given, the multigoal path planning problem can be directly formulated as the TSP [24]. In general, a determination of such a collision-free path for a mobile robot can be computationally very demanding [25]. However, if a point robot can be assumed and the robot workspace can be represented by the polygonal domain, the shortest path roadmap approach can be used [26]. Thus, a solution of the TSP in a form of the found tour, for example, using visibility graph, can be considered as the requested collision-free path (solution) of the MTP for a single mobile robot.

In robotic planning, cities can represent sensing locations at which the robot gathers information about its surrounding environment to “see” the whole workspace [27]. The problem of searching the workspace is called the inspection task, and one of the feasible approaches is based on a formulation as a problem of finding the set of sensing locations and consecutive solution of the TSP [28]. Suitable sensing locations can be found by a sensor placement algorithm, for example, [29–32]. Then, a group of cooperating mobile robots can be used to decrease the required time to inspect the environment and thus the inspection task can be formulated as the MTSP [20].

Several methods for the MTSP have been proposed in literature which is also the case for a very closed problem formulation known as the Vehicle Routing Problem (VRP) where capacity of each vehicle is considered [19]. Beside auction-based techniques [33] and multiagent solutions [34], soft-computing techniques such as genetic algorithms have been proposed for these problems [35]. Note that the MTSP can be transformed into the TSP using transformation proposed in [36]; however, such a solution can be highly degenerated for the MTSP with minmax objective. It is because, in the TSP, the total tour length is minimized, that is, a tour with zero length can be provided while a sum of the lengths of the all tours for individual salesmen can be minimal. Therefore, it is necessary to address the minmax objective directly [37].

The MTSP-Minmax has been addressed by the combinatorial heuristic in [22], where authors propose to find optimal solution of the MTSP-Minmax using the distance constraint VRP formulation. A solution of the MTSP is used as the distance constraint that is gradually decreasing and if the VRP does not have a solution, the previous solution of the MTSP is considered as the optimal solution.

Soft-computing techniques have been also applied to the MTSP-Minmax, such as ant colony optimization [38], genetic algorithms [39], and also SOM in [16]. Particular soft-computing approaches for Euclidean instances of the MTSP-Minmax have been evaluated in [40] and our early results on robotic problems with obstacles in [41]. Therefore, in the presented work, we are focused on the evaluation of the SOM-based solution of the multirobot multigoal path planning and its comparison with the GENIUS algorithm [22]. The used SOM is directly based on [16] combined with the ideas proposed in [41] that have been accompanied by the approximation of the shortest path originally proposed in [18, 21], which significantly decreases the required computational time for SOM adaptation. Therefore, GENIUS [22], the considered SOM [16], and the approximations of the shortest path are described in detail in Section 4.

## 3. Problem Statement

The studied problem of the multirobot multigoal path planning with minmax objective is motivated by inspection missions, where a group of mobile robots is requested to visit a given set of sensing locations, where sensor measurements are taken. In particular, the mission is to inspect all reachable areas of the environment as quickly as possible. The environment is represented by a polygonal map and the given sensing locations are determined in such a way that the whole environment is covered by visiting them [32]. It is assumed that a map of the environment is available; each robot has a differential drive and its shape can be bounded by a disk with a limited radius. For simplicity and without loss of generality, a point robot is considered in the polygonal domain *𝒲* created by enlarging the original map by the radius of the disk, and all free space is reachable by the robot. Then, the multigoal path planning problem is formulated as the MTSP-Minmax that can be defined as follows:* for a given polygon with holes 𝒲, a set of cities (sensing locations)* **C** *lying inside 𝒲 and k salesmen (robots) find k closed tours starting at the selected city c*_d_ ∈ **C** *such that each city c* ∈ **C**∖{*c*_d_}* is visited by one salesman and the length of the longest tour is minimized.* The city *c*_d_ is called the depot in the rest of this paper.

### 3.1. Used Notation

The SOM adaptation schema is considered in the polygonal domain *𝒲*; therefore, few terminology notes are presented here to clarify the used terms and symbols for underlying geometrical structures utilized in the approximation of the shortest path in *𝒲*.

The robot workspace is represented by the polygonal map *𝒲* consisting of *v* vertices and thus *𝒲* is a closed, multiply connected region, whose boundary is a union of *v* line segments, forming *h* + 1 closed polygonal cycles (polygons), where *h* is the number of holes (obstacles). A distance between two points inside *𝒲* is a length of a path among obstacles that can be a straight line segment or consists of the map vertices. Thus, a path between two points *s* and *t* consists of a finite number of straight line segments joining the points and vertices of *𝒲*.

*𝒲* can be divided into a set of nonoverlapping convex polygons that are formed from vertices. Such convex polygons are called cells and represent* convex polygon partition* of *𝒲*; that is, each cell *C* forms a closed polygonal cycle of line segments joining vertices. A line segment is called* diagonal* if it connects two nonadjacent vertices and it is entirely contained in *𝒲*. A point inside *𝒲* is always inside some cell and a path between two points *s* ∈ *C*_*s*_ and *t* ∈ *C*_*t*_ can be constructed from the shortest path between vertices of *C*_*s*_ and *C*_*t*_.

Regarding SOM for the TSP, weights of a particular neuron represent a point *ν* (called node) that lies in *𝒲* and therefore *ν* is always inside some cell. Such a cell of the node *ν* is denoted as *C*_*ν*_. An overview of the used symbols is in Symbols section at the end of the paper.

### 3.2. Quality of Solution

The motivational problem of the multigoal path planning for a group of cooperating robots is formulated as the MTSP. The minmax variant of the MTSP leads to minimizing the longest tour and therefore we consider the maximal length *L* of the individual tours {*l*_1_,…, *l*_*k*_} as one of the solution quality indicators. However, SOM and also GENIUS are randomized algorithms and therefore the performance indicators should be computed from several trials. For the TSP, the usual indicators are the percentage deviation of the mean solution to the optimum tour (denoted as the PDM) and the percentage deviation from the optimum of the best solution value, denoted as the PDB. Finding an optimal solution for the considered instances of the MTSP-Minmax is computationally very demanding and therefore the best found solution for the particular problem instance (found by the evaluated algorithms) is considered as the reference solution. The longest tour of this reference solution is denoted as *L*_REF_ and it is used to compute the PDM and PDB as follows:

(i) Reference solution length *L*_REF_ of the particular problem instance found as the longest tour of the best solution of several solutions found by particular selected algorithm(s).
(ii) Maximal length of the individual tours in a solution of the MTSP. Consider
  (1) $$\begin{array}{c} L=\max\left\{\;l_{1},\ldots ,l_{k}\;\right\}. \end{array}$$
(iii) Percentage deviation to the reference solution value *L*_REF_ of the mean solution value $\bar{L}$. Consider
  (2) $$\begin{array}{c} PDM=\frac{\bar{L}-L_{REF}}{L_{REF}}\cdot 100\%. \end{array}$$
(iv) Percentage deviation to the reference solution value *L*_REF_ of the best solution value *L*_BEST_. Consider
  (3) $$\begin{array}{c} PDM=\frac{L_{BEST}-L_{REF}}{L_{REF}}\cdot 100\%, \end{array}$$
 where *L*_BEST_ is the best (the shortest the longest tour) solution from several solutions of the particular problem instance found by a particular algorithm variant.

The advantage of the percentage deviations of the tour lengths is that they provide a scale independent metric for particular instances of the MTSP and thus it can be used to aggregate results for various problems and many trials. However, it does not provide any indication to how the workload is divided into the particular robots; that is, what are the differences in the lengths of the individual tours? We propose two quality indicators to measure the quality of cooperation. The first is a percentage deviation of the lengths in a tour. This indicator is called a* Cooperative Quotient (CQ)* and its zero value means an* ideal cooperation*. The second indicator considers the total travelled distance by all robots and it is called* Collaborative Effort (CE)*. These indicators are computed as follows:

(i) Cooperative Quotient is compouted as
  (4) $$\begin{array}{c} CQ=\frac{s_{L}}{\bar{L}}, \end{array}$$
 where *s*_*L*_ is the root of the sample variance, $s_{L}^{2}=(1/(k-1))\sum_{i=1}^{k}(L_{i}-\bar{L}{)}^{2}$, and $\bar{L}$ is the average value of the tour lengths.
(ii) Collaborative Effort is computed as
  (5) $$\begin{array}{c} CE=\overset{k}{\underset{i=1}{\sum }}l_{i}. \end{array}$$

## 4. Use Approaches

### 4.1. GENIUS

The GENIUS algorithm has been used to find a solution of the MTSP-Minmax in [22]. It is a combinatorial method representing a general approach for the TSP that is based on two heuristics: GENI (Generalized Insertion) and US (Unstringing and Stringing) [42]. The first heuristic is a construction method while the second heuristic is an optimization method. Tours are initially constructed by GENI. After that, the tabu search technique is used to exchange cities from one tour to another, while GENI is utilized for vertices inserting/removing. Finally, the US optimization procedure is used. It removes a vertex from the tour and inserts the vertex into the same tour by GENI. The procedure is repeated until a vertex reinsertion improves the quality of solution. The parameter *p* of the GENI algorithm defines the size of the neighborhood that is used to select the best possible vertex insertion. Performance of the tabu search can be controlled by three additional parameters: *q*, Θ, and *T*_max_. The *q* parameter determines the size of the global neighborhood to select an appropriate tour for a vertex exchange and Θ controls the number of iterations for which a move of vertex according to the particular tour is declared tabu. The maximum allowed number of iterations without improvement is defined by the *T*_max_ parameter.

Recommended values of parameters have been suggested by authors [22]. Two sets of parameters can be considered. The first set (*p* = 5, *q* = 5, *T*_max_ = 10) can be called* fast*, because it provides a compromise between computational requirements of the algorithm and the quality of solution. The second set (*p* = 14, *q* = 5, *T*_max_ = 100) provides high quality solutions, but it is computationally demanding. That is why the algorithm with this set of parameters is denoted as GENIUS-*quality* in this paper. Note that for each operation stored in the tabu list, the value of Θ is selected randomly from the interval 〈7,27〉.

GENIUS is a combinatorial approach; therefore, only distances between cities are need. In the case of the Euclidean TSP, distances can be computed as the Euclidean distance, while for the motivation problem of multigoal path planning, shortest paths between cities have to be found. The shortest paths can be determined from the full visibility graph that can be constructed in *O*((*v* + *n*)^2^) [43], where *v* is the number of vertices of *𝒲* and *n* is the number of cities. All shortest paths between cities can be found by Dijkstra's algorithm in *O*(*ne*log⁡(*v* + *n*)), where *e* is the number of edges of the visibility graph. All distances of the shortest paths can be precomputed and stored in the distance matrix.

### 4.2. SOM Adaptation Schema for the MTSP-Minmax

**Figure 1:**
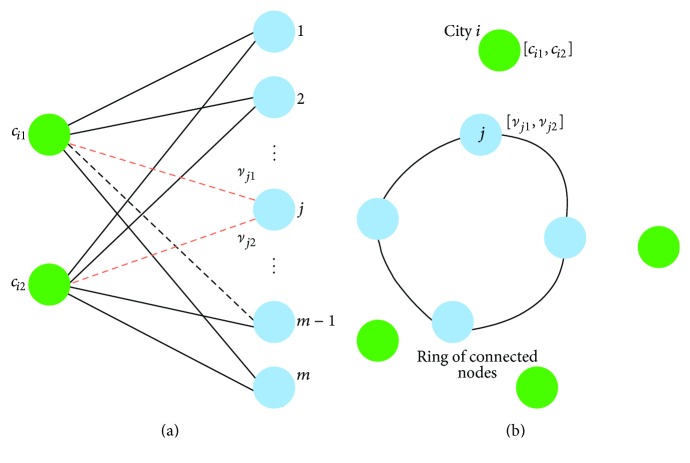
Schema of the SOM two-layered neural network for the TSP and associated geometric representation.

The SOM for the MTSP-Minmax [16] uses two-layered competitive learning networks, where each network contains two-dimensional input vector and an array of output units. An association between the learning network and its geometrical representation of one TSP tour is shown in Figure 1. An input vector *i* represents coordinates (*c*_*i*1_, *c*_*i*2_) of the city *c*_*i*_ and weights *ν*_*j*1_ and *ν*_*j*2_ can be interpreted as coordinates of the node *ν*_*j*_. Nodes are connected to a ring representing the tour; thus, an individual ring of nodes is created for each salesman. The network is initialized with small random connection weights and cities are then sequentially applied to the network in a random order to avoid local minima. The output nodes compete to be the winner for a given city according to the following competitive rule:

(6) $$\begin{array}{c} \nu^{*}=\underset{\nu }{\arg\;\min}\left|\;c,\nu \;\right|\cdot \left(\;1+\frac{l_{\nu }-l_{avg}}{l_{avg}}\;\right), \end{array}$$

where |*c*, *ν*| denotes the Euclidean distance between the city *c* and the node *ν*, *l*_*ν*_ is the length of the ring, into which the node *ν* belongs, and *l*_avg_ is the average length of the rings. Basically, the rule prefers nodes from shorter rings and thus it aims to minimize the longest ring (tour).

The weights of the winner node and its neighbouring nodes are updated to get closer to the presented city according to the neighbouring function *f*(*G*, *d*). The adaptation function moves a node *ν*_*j*_ towards the city *c*_*i*_ by the rule

(7) $$\begin{array}{c} \nu_{j}^{\prime }=\nu_{j}+\mu f\left(\;G,d\;\right)\left(\;c_{i}-\nu_{j}\;\right), \end{array}$$

where *μ* is the fractional learning rate. The used neighbouring function is *f*(*G*, *d*) = exp⁡(−*d* ^2^/*G* ^2^) for *d* < 0.2*m* and *f*(*G*, *d*) = 0 otherwise, where *G* is the gain parameter, *d* is the distance (in the number of nodes) of a node from the winner measured along the ring, and *m* is the number of nodes in the ring that is set to *m* = 2.5*n*/*k*, where *n* is the number of cities and *k* is the number of salesmen. The gain *G* is decreased after each complete presentation of the cities to the network (one learning epoch) according to the gain decreasing rate *α*. An appropriate initial value of *G* depends on the number of cities *n* and it is set according to the formula *G*_0_ = 0.06 + 12.41*n*. The used values of learning and decreasing rates are *μ* = 0.6 and *α* = 0.1 [16], respectively. The whole adaptation procedure of the SOM for the MTSP-Minmax is depicted in Algorithm 1.

In the MTSP with a common depot, the adaptation procedure must ensure that all tours are connected with the depot. Therefore, a winner node from each ring is selected and adapted to the depot. After that, other cities are presented to the network in a random order and the winner node is selected from all noninhibited nodes. The network evolves until each city has the winner node sufficiently close.

**Figure 2:**
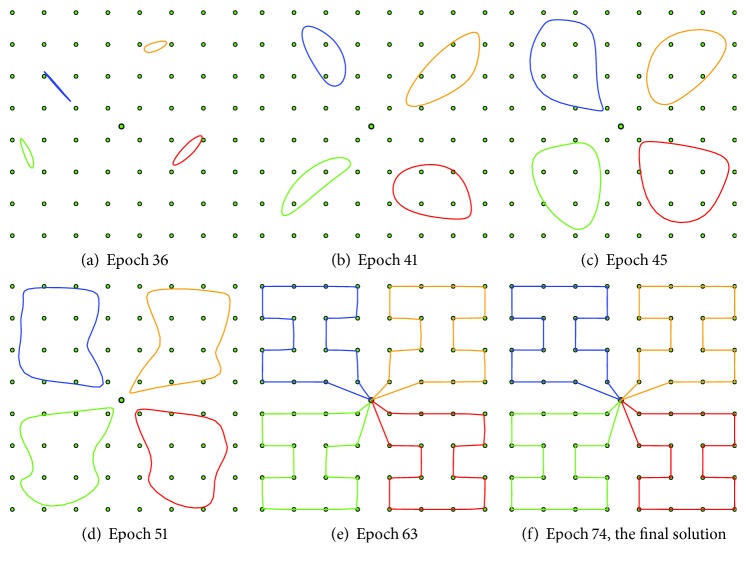
An example of SOM [16] evolution at the particular learning epochs in an instance of the MTSP-Minmax without obstacles.

An inhibition mechanism is used to associate distinct winners to each city during one learning epoch, that is, a complete presentation of all cities to the network. A winner node is marked as inhibited and it does not compete to be winner for another city for the rest of the current learning epoch. At the end of each epoch, tours can be constructed from the winners by traversing each ring. The length of each tour can be then found as a sum of the city–city distances. An example of the algorithm performance for the Euclidean MTSP-Minmax is shown in Figure 2.

The efficiency of the SOM algorithm relies on the determination of the winner (6), which uses a node–city distance. Moreover, the MTSP-Minmax needs an efficient determination of the shortest path between two nodes (node–node) to compute the length of each individual ring. The winner is then adapted towards the city, which can be interpreted as a movement along the shortest path to the city according to the neighboring function *f* in (7). In the multigoal path planning problem, nodes have to be inside *𝒲* and therefore all paths (and distances) have to respect obstacles. The efficient determination of the collision-free path in a presence of obstacles is therefore crucial for an applicability of the SOM procedure to the robotic planning problems.

### 4.3. Approximation of the Node–City Path

**Figure 3:**
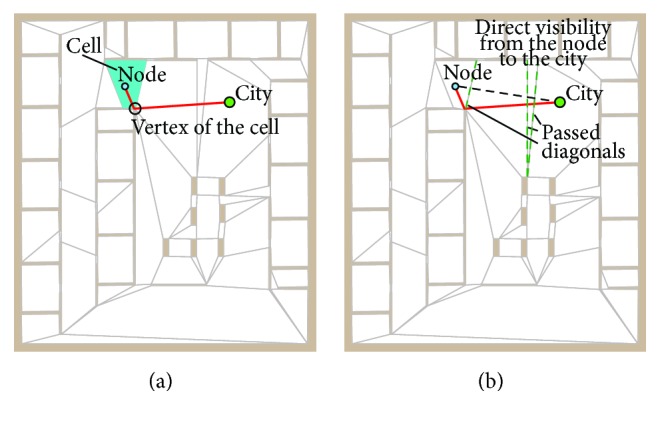
An example of approximate shortest path and passed diagonals.

The idea of the quick determination of the collision-free path in *𝒲* has been proposed in [18] and it is based on determination of approximate path in a supporting division of the free space into convex cells that forms a convex polygon partition. The convex polygon partition is induced by diagonals; therefore, each cell of the convex partition consists of diagonals and edges representing obstacles or the border polygon of *𝒲*. During the SOM adaptation, a node (neuron weights) is always placed inside *𝒲* and thus it is always placed in some convex cell of the partition. The shortest path from a vertex of such a cell to the particular city can be used as approximation of the shortest path from a node to the city. Note that such a path passes diagonals of the convex polygonal partition (see example in Figure 3) which is further utilized to improve the approximation.

The shortest paths from map vertices to all cities can be found in the visibility graph, for example, by Dijkstra's algorithm in time *O*(*ne*log⁡(*v* + *n*)), where *n* is the number of cities, *v* is the number of vertices, and *e* is the number of visible pairs (city–city, city–vertex, and vertex–vertex), which can be bounded to *e* ≤ *v* + *vn*. The graph can be found in *O*((*v* + *n*)^2^) using the algorithm [43].

The approximation can be formally described as follows. Let a polygonal representation of the robot workspace be *𝒲* with *v* vertices and let **P** be a convex polygon partition of *𝒲* into convex cells *C*_*i*_, **P** = {*C*_1_, *C*_2_,…, *C*_*r*_}, where each cell *C*_*i*_ is represented as a sequence of polygon vertices and a node *ν* is in a cell *C*_*ν*_. The initial approximate path from *ν* to the city *c* is found as the shortest path *𝒮*(*w*, *c*) over vertex *w* of *C*_*ν*_ to *c* such that *w* = arg min_*w*_*i*_∈*C*_*ν*__⁡|*ν*, *w*_*i*_| + |*𝒮*(*w*_*i*_, *c*)|, where |·, ·| denotes the Euclidean distance between two points and |*𝒮*(·, ·)| is the length of the shortest path between two vertices (vertex and city in particular). The problem of finding the cell *C*_*ν*_ is the point-location problem, which can be solved in *O*(log⁡*v*) or in the average complexity *O*(1) by the “bucketing” technique [44].

Such a rough approximation of the shortest path can be further improved by an iterative evaluation of the direct visibility from the node to the vertices of the approximate path. Let a node *ν* be inside the cell *C*_*ν*_ and approximation of the path from *ν* to the vertex *v*_*l*_ be a sequence of vertices (*v*_0_, *v*_1_,…, *v*_*l*_), *v*_0_ ∈ *C*_*ν*_. Then, the refinement of the path is an iterative examination of the direct visibility test between *ν* and *v*_*i*_ that iterates over the particular vertices of the path. The visibility test is based on the method described in [45]. Just instead of a triangulation used in [46], we propose using a convex partition. If a straight line from *ν* to the vertex *v*_*j*_ for 0 < *j* < *l* crosses only diagonals or it entirely lies in one cell, then the vertex *v*_*j*_ is directly visible and all vertices *v*_*i*_ for *i* < *j* can be removed from the sequence representing the collision-free path from *ν* to *c*; see Figure 3 where a direct connection of the node with the city passes only diagonals and thus it is a collision-free.

**Figure 4:**
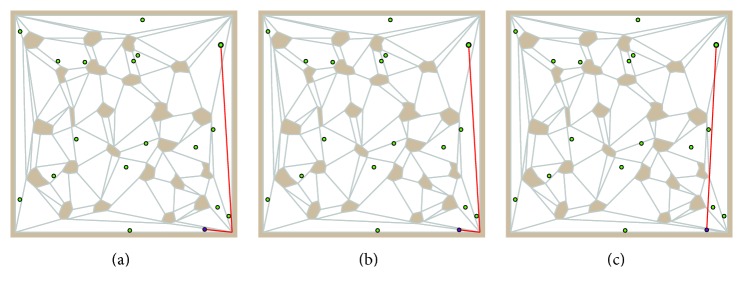
An example of the path approximation and its improvement during the adaptation: (a) node and city are not directly visible; (b) after the node movement towards the city, (c) the city becomes directly visible and the path refinement decreased the length about ten percent.

The complexity of this path refinement depends in the worst case on the number of vertices and it can be even worse than a determination of the visibility graph from *ν*. However, the real-time performance is much better [18]. For example, if only one direct visibility test is considered, then, in final learning epochs, nodes are very close to the cities; hence, the node is in the same cell as the city or it is just in the next cell. Also if a node and the city are not directly visible, after the node movement towards the city along the approximate path, the city becomes visible and the path refinement provides the shortest path; see Figure 4. This expected behaviour has been experimentally verified for particular variants of the path refinement and various environments in [18].

### 4.4. Approximation of the Node–Node Path

In the SOM for the MTSP-Minmax, it is also necessary to determine the lengths of the particular rings that represent the individual tours for the robots (salesmen). In this case, node–node distances have to be computed, which represent two-point shortest path queries. Here, precomputed shortest paths from the map vertices to the cities do not help. However, approximation of the shortest path between two nodes can be based on the algorithm for the approximate the node–city path. This idea has been utilized in [21] to compute the coverage of *𝒲* from the current ring. The approximation works as follows.

Let a node *ν*_1_ be in the cell *C*_1_ and a node *ν*_2_ be in the cell *C*_2_. A path between *ν*_1_ and *ν*_2_ is constructed from the shortest path between vertices of each cell *𝒮*(*w*_1_, *w*_2_), where *w*_1_ ∈ *C*_1_ and *w*_2_ ∈ *C*_2_. The particular vertices *w*_1_ and *w*_2_ are selected according to the minimization of the total path length |*ν*_1_, *w*_1_| + |*𝒮*(*w*_1_, *w*_2_)| + |*w*_2_, *ν*_2_|. Such a path can be refined in a similar manner like the aforementioned node–city path. For a further details, see [20, 21].

In this approximation, only the convex partition and the visibility graph for the vertices of *𝒲* are utilized. The visibility graph for the cities is not used; therefore, the needed supporting structures depend only on *𝒲*. Note that for a very high number of cities, for example, several thousands, this approximation of the node–node shortest collision-free path can be utilized also for the node–city distance queries in the winner selection and thus it can reduce the total memory requirements of the algorithm.

## 5. SOM for the MTSP-Minmax in the Polygonal Domain

Having the approximations of the shortest node–city and node–node paths, the SOM for the MTSP–Minmax [16] (described in Section 4.2) can be directly applied to multigoal path planning problems in the polygon domain *𝒲*. The main difference is that instead of the Euclidean distance, the needed distances in the adaptation are computed as the length of the collision-free path found by the described approximations. Thus, paths found by the approximations of the shortest node–city and node–node paths are used in Algorithm 1 in the  select_winner  and  adapt  procedures.

**Figure 5:**
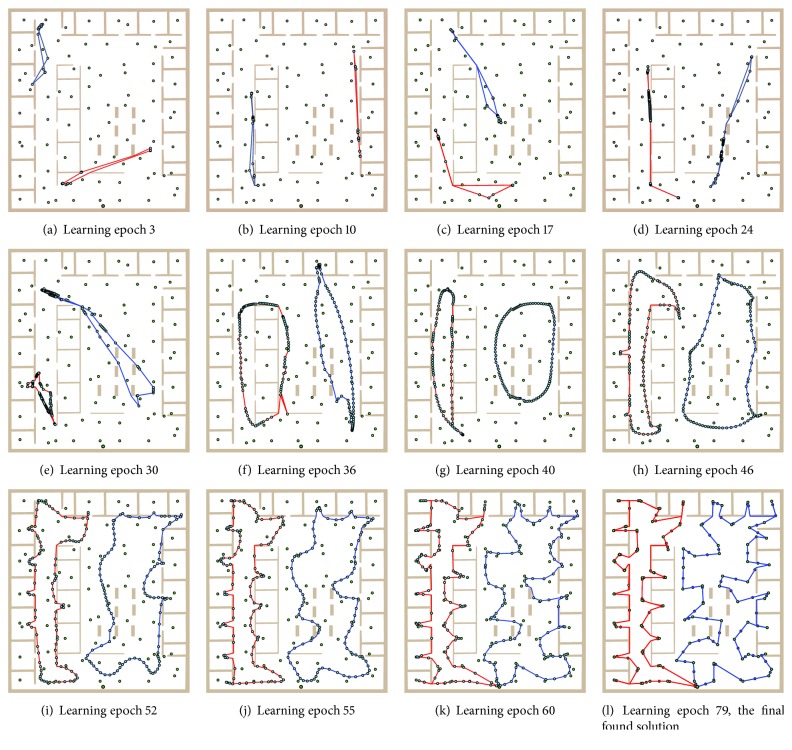
An example of SOM evolution in *𝒲*; small standalone disks represent cities and connected disks represent nodes that form rings and finally the tours.

For the adaptation phase, the paths are considered for updating the neuron weights such that the weights are set to represent a point at the particular straight line segment of the path. Just these distances and movement of the nodes in the adaptation are changed in the SOM schema. All other parts and properties remain from the original algorithm [16]. An example of the SOM evolution in *𝒲* is shown in Figure 5.

Beside the approximation of the node–node path, a length of the ring can be estimated in a less computationally intensive way by the following approach. The ring of nodes represents a tour over cities and the length of the tour can be used as the ring length to compute the weight in the competitive rule (6). If shortest distances between the cities are precomputed, such a length can be determined in a linear time (proportionally to the number of cities in the particular tour represented by the ring). It is only necessary to maintain association of the city with its winner in the current learning epoch. The city tour is formed from the cities associated with the winners. However, the winner is associated with the city until a new winner is selected. If a node has been selected as a winner to one city in the previous learning epoch and as a winner to another city in the current epoch, the association with the previous city is cleared to reflect change of the ring shape. A tour represented by the ring is then formed by the cities associated with the nodes along the ring.

Note that particular winners can be pulled away from the cities during adaptation of another winner, because they can be in its neighborhood. So, such a city–city tour is only approximation of the current ring. Tours represented by the rings may not necessarily contain all the cities. For example, in the initial phase of the adaptation, only the cities presented to the network can have their winner. Thus, the length of the tour can be a rough approximation of the ring length. However, in the final learning epochs, most of the winners are preserved over the epochs and this approximation of the ring length is becoming more accurate.

**Figure 6:**
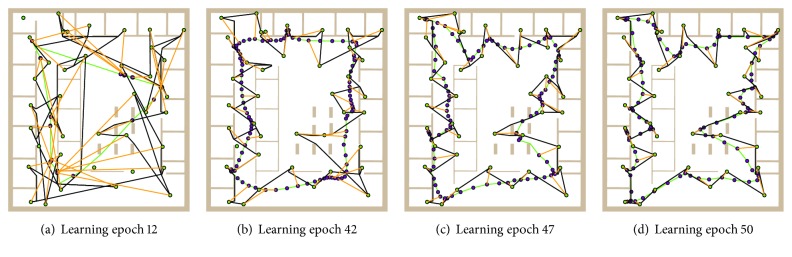
Examples of tour represented by the ring; the final solution is found in 72 learning epochs.

Examples of city–city tour represented by the current ring are shown in Figure 6, where only one salesman is considered for illustrative purposes. The first figure shows tour after eleven complete presentations of all cities during the twelfth learning epoch. Although the ring does not contain self-crossings, the tour has several self-crossings and it does not visit all the cities. After several learning epochs, the tour is complete and finally it is the same as the ring, because winners match the cities.

## 6. Results

The impact of the proposed approximations of the shortest paths in *𝒲* to the solution quality and computational requirements of the SOM for the MTSP-Minmax [16] has been evaluated in several multirobot multigoal path planning problems motivated by inspection missions. Due to a lack of common instances of the MTSP for environments with obstacles, a set of environments used in the motion planning has been utilized (maps and all the evaluated problems are available at http://comrob.fel.cvut.cz/jf/data/mtsp/). For these environments, cities have been found as a set of sensing locations in the inspection task, for example, like in [27], by the sensor placement algorithm [32].

Parameters of the environments are shown in Table 1, where *v* is the number of vertices, *h* is the number of holes, and *r* is the number of convex cells (regions) of the supporting convex partition. Environments* jh*,* pb*,* ta*, and* h2* represent maps of real buildings; thus, they provide a representative size of the inspection planning problems. The examined problems are organized into three sets (see Table 2), where *n* denotes the number of sensing locations (cities) and the subscript in the name denotes visibility range in meters utilized for the sensor placement; see [32].

Beside the sensing locations considered as the cities, a particular location of the depot also influences a solution of the MTSP. The depot has been placed as an additional city in the free space that is close to the center of the free space of *𝒲*. In addition, for* warehouse*,* jh* and* h2* environments, the depot is also placed near to the entrance and therefore two problems are created for these environments. The subscripts* A* and* B* are used to distinguish position of the depot, where* B* denotes the depot close to the entrance.

The performance of the SOM algorithm with the proposed shortest path approximations is compared with solutions found by the GENIUS algorithm for both parameters as the GENIUS-*quality* and GENIUS-*fast* variants; see Section 4.1. The SOM for the MTSP-Minmax is considered in two variants according to the utilized method to determine a length of the ring. The first variant is based on the approximation of the shortest node–node path described in Section 4.4 and it is denoted as SOM-*nn*. The second variant, called SOM-*cc*, uses a length of the city tour represented by the ring; see Section 5.

Both SOM and GENIUS are randomized algorithms; therefore, each problem has been solved 20 times by the particular algorithm variant. The used notation follows Section 3.2, but ratios are used rather than absolute values for presenting aggregated results among the particular set of problems according to Table 2.

The parameters of the SOM procedure are used as they have been presented in Section 4.2. The adaptation has been terminated if the *error* is less than 0.001 or after 180 learning epochs. A number of neurons in the ring is set to 2.5*n*/*k*, where *n* is the number of cities and *k* is the number of salesmen. The utilized node–city approximation uses the full path refinement (*pa*); see [18] for further details.

### 6.1. Aggregated Results for the Problem Sets

For aggregated results, LR denotes the average length ratio *L*/*L*_REF_, where *L* is the length of the longest tour and *L*_REF_ is the best solution found by the GENIUS-*quality*. The average ratio of CE is computed as the ratio of the particular CE and the value of CE for *L*_REF_; the ratio is denoted as CER. The average value of CQ is used, because it is already relative for the particular solution. The required computational time of the path refinement is evaluated as the time ratio TR. It is computed as the time to find a solution divided by the average required time for the same problem and selected algorithm variant. Standard deviations are computed as the root of sample variances.

*GENIUS Heuristic*. Performance of the GENIUS algorithm for the* quality* and* fast* variants is presented in Table 3. The standard deviations of LR are about 0.06 for all presented results. The reference value *L*_REF_ for LR is found as the best solution of GENIUS-*quality*. In both variants, CQ is very low; therefore, found solutions have almost identical length to the tours. Values of CER are higher than those in all cases. It is mainly due to the postoptimization procedure, which is repeated only if the longest tour is shorter than other tours after the US postoptimization procedure. The algorithm is terminated if the shortened tour is still the longest tour. A further improvement of other tours can be possible, but it will not decrease the minmax objective.

*Determination of the Ring Length*. Aggregated results for the SOM-*nn* and SOM-*cc* variants are presented in Table 4. Also in this case, the reference value for LR is found as the best solution of GENIUS-*quality*, but the reference value for the time ratio TR is the required computational time for the SOM-*nn* algorithm variant. The standard deviations of LR values are about eight percent. In both SOM variants, the solution is found in less than 86 and 100 learning epochs for the* middle* and* large* problem sets, respectively. Here, the proposed *cc* ring length determination in SOM-*cc* outperforms the SOM-*nn* variant in the solution quality and also in the required computational time. Note that CER is less than one. Even though LR is lower about few percentage points for SOM-*cc*, CER is almost identical for both variants. CQ increases with the number of salesmen, which indicates higher differences in the individual tour lengths of the particular solution.

Results presented in Tables 3 and 4 provide an overall comparison of the algorithms' performance. Regarding values of LR, the GENIUS algorithm provides overall better solutions with respect to the minmax objective. For the* large* set, the SOM-*cc* variant provides similar LR like GENIUS-*quality* and significantly better results than GENIUS-*fast*. The CER is lower for SOM, which is mainly because GENIUS improves only the longest tour.

### 6.2. Results for Individual Problems

Particular results for individual problems for three salesmen (*k* = 3) and five salesmen (*k* = 5) are presented in Tables 5 and 6, respectively. Here, *L*_REF_ denotes the length (in meters) of the longest tour of the best found solution from all solutions found by the four evaluated algorithm variants. The columns PDM and PDB denote the particular percentage deviations from *L*_REF_, namely, Section 3.2.

**Figure 7:**
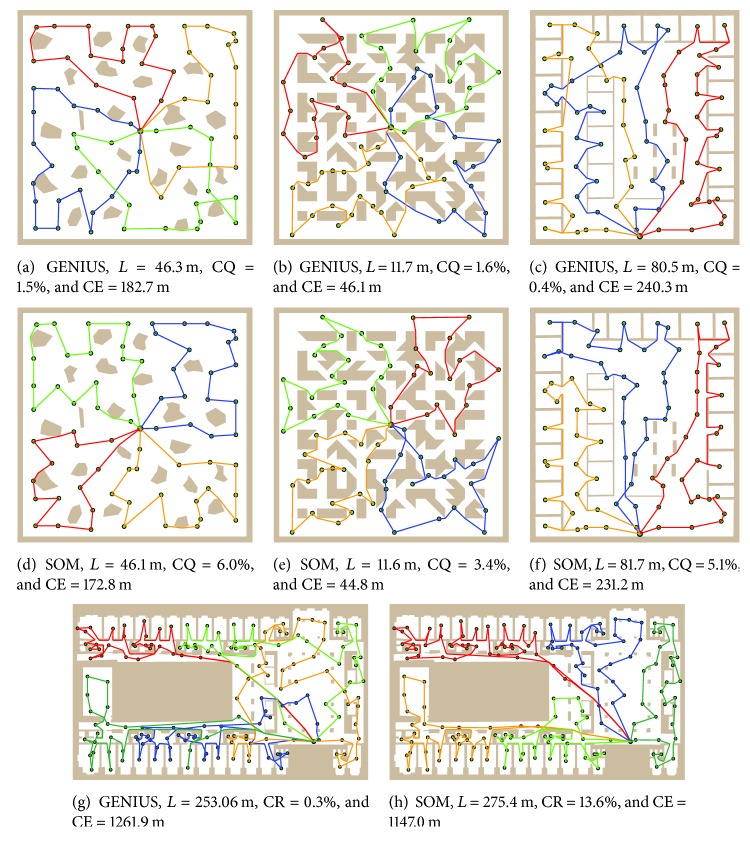
Examples of found solutions by the GENIUS-*quality* and SOM-based algorithm. *L*: length of the longest tour, CR:* Cooperative Ratio*, CE:* Collaborative Effort.* (a, d)* potholes*, four salesmen; (b, e)* m3*, four salesmen; (c, f)* jh*, three salesmen; (g, h)* h2*, five salesmen.

Overall, solutions found by the GENIUS-*quality* are better according to the PDM and PDB. However, an important aspect of the SOM solutions should be remarked. SOM tries to preserve a topology of the input space, which leads to preferring solutions without mutually crossings tours. This behaviour is illustrated in the selected four best solutions found by the GENIUS and SOM algorithms that are presented in Figure 7. Note that both algorithms found solutions with very similar length to the length of the longest tour. From the path planning point of view, the SOM solutions provide an interesting feature, because if found tours do not cross, such a solution also automatically guarantees the coordination of the robots motion.

### 6.3. Real Required Computational Time

The real required computational time of the evaluated GENIUS and SOM algorithms has been measured during the experimental verification. Two supporting structures have to be precomputed for the used approximation of the shortest paths in SOM: the polygon partition and the visibility graph. The required time to create a convex polygon partition is in units or tens of milliseconds and it is negligible in comparison to the required time of the SOM adaptation procedure. Also the construction of the visibility graph is very fast in comparison to SOM or GENIUS algorithms. It is found in 41 milliseconds for the largest problem with 575 cities. The most time expensive part of the preparation phase is the computation of the shortest paths between cities (and vertices); this is required for both GENIUS and SOM algorithms. Therefore, this time is included in the presented results.

The algorithms have been implemented in C++ and compiled by G++ 4.2 with -O2 optimization. All results presented in this paper have been computed using the same computational environment, with the Athlon X2 CPU running at 2 GHz and 1 GB RAM and only one CPU core has been utilized. Therefore, real computational requirements of each particular algorithm can be directly compared with the results presented in Tables 5 and 6.

**Figure 8:**
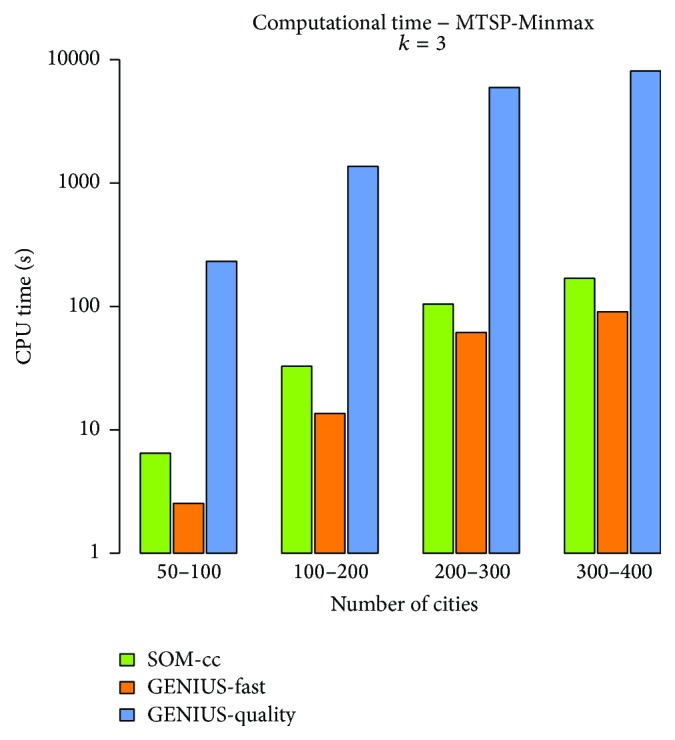
Required computation time.

The computational time depends on the number of cities and also on the particular environment; therefore, times can be presented as histograms of average values for a range of the number of cities. The average required computational time for instances of the MTSP with three salesmen is presented in Figure 8. The GENIUS-*quality* algorithm is very computationally intensive, while GENIUS-*fast* is faster than the proposed SOM-*cc*. According to the quality of found solutions, the SOM-*cc* provides the best trade-off between the quality of solutions and the required computational time.

## 7. Conclusion

The SOM adaptation procedure for the MTSP-Minmax has been applied to problems in the polygonal domain, which represent instances of the non-Euclidean MTSP. The motivation of the studied problem is multigoal path planning in the polygonal domain; thus, the problem remains in a plane. However, the main issue of SOM in this type of problems is a determination of the shortest path among obstacles, which is needed in the competitive and adaptation phases of SOM for the TSP. Therefore, a fast determination of the shortest path is needed, which can be addressed by approximate path and its determination is supported by suitable data structures. The used approach is based on the convex partition that (according to the results) provides a sufficient quality of the approximation while it is also computationally feasible.

The proposed algorithm has been performed more than five thousand times, which indicates its sufficient robustness. The experimental results also show that the proposed SOM-*cc* variant provides better solution than a direct computation of the ring length. SOM-*cc* does not require approximation of the shortest path between two nodes; therefore, only the node–city path queries are part of the adaptation procedure. The quality of found solutions is competitive with the general heuristic GENIUS, but the SOM algorithm is less computationally intensive than the GENIUS-*quality* variant.

The presented work in this paper is based on an experimental application of well known and an already available SOM adaptation schema. Based on the experiments, the following results can be considered as the main contributions of the paper:

(i) Relatively simple supporting structures allow application of SOM principles in the polygonal domain.
(ii) SOM provides interesting feature for the multirobot path planning, because solutions with noncrossing tours are advantageous for a multirobot coordination.
(iii) SOM-based algorithm provides competitive results to the combinatorial algorithm GENIUS in the examined instances of the non-Euclidean MTSP-Minmax in the polygonal domain.
(iv) A rough approximation of the shortest node–city paths seems to be sufficient for the SOM adaptation procedure, which enables possible SOM application in 3D multigoal path planning, where approximation of the shortest path is necessary.
(v) A length of the ring can be computed by a length of the tour represented by the ring, which avoids necessity of two-point shortest path queries.

Even though the proposed approach provides competitive results for the examined non-Euclidean MTSP, it can be improved in several ways. First, the used SOM schema [16] will be more likely outperformed by more recent SOM variants, for example, by the Coadaptive Net algorithm [10], which uses restricted set of nodes in the winner node selection phase and also less number of neighbouring nodes to a winner node can be adapted. Thus, the computational time can be further decreased. The approximations of the shortest path can be used in other SOM-based algorithms for the TSP, because it principally provides the distance between a node and the presented city to the network.

Regarding the found feature of SOM solutions of the MTSP, the noncrossing tours are more likely found, and a frequency of such solutions guaranteeing multirobot coordination can be increased. In [47], authors proposed an adaptation procedure for two robotic arms, which can be possibly applied in the MTSP. From this perspective, SOM should pay more attention to planning problems where both the cooperation and the coordination are part of the planning.

## Figures and Tables

**Algorithm 1:**
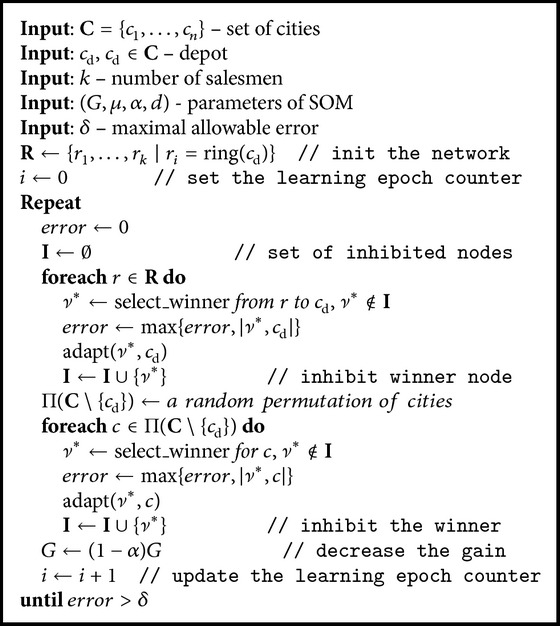
SOM for the MTSP-Minmax.

**Table 1:** Testing environments with obstacles.

Name	Dimensions [m × m]	Area [m^2^]	*v*	*h*	*r*
jari	4.5 × 4.9	20	48	1	14
complex2	20.0 × 20.0	322	40	3	21
m1	4.8 × 4.8	20	51	4	26
m2	4.8 × 4.8	15	51	6	20
map	4.8 × 4.8	14	68	8	36
potholes	20.0 × 20.0	367	153	23	75
rooms	20.0 × 20.0	351	80	0	33
a	8.9 × 14.1	71	99	6	22
dense	21.0 × 21.5	299	288	32	150
m3	4.8 × 4.8	17	308	50	120
warehouse	40.0 × 40.0	1192	142	24	83
jh	20.6 × 23.2	455	196	9	77
pb	133.3 × 104.8	1453	89	3	41
ta	39.6 × 46.8	731	74	2	30
h2	84.9 × 49.7	2816	2062	34	476

**Table:** (a) Small set

Problem	*n*
jari	6
complex2	8
m1	11
m2	12
map	18
potholes	18
rooms	21
a	22

**Table:** (b) Middle set

Problem	*n*
dense_4_	53
potholes_2_	68
m3_1_	71
warehouse_4_	79
jh_2_	80
pb_4_	105
ta_2_	141
h2_5_	168

**Table:** (c) Large set

Problem	*n*
potholes_1_	282
jh_1_	356
pb_1.5_	415
h2_2_	568
ta_1_	574

**Table:** (a) Problem set *middle*

*k*	GENIUS-*quality*			GENIUS-*fast*		
	LR	CQ	CER	LR	CQ	CER
2	1.04	0.003	1.04	1.07	0.003	1.07
3	1.06	0.005	1.06	1.11	0.006	1.11
4	1.06	0.007	1.06	1.14	0.011	1.14
5	1.06	0.010	1.06	1.17	0.015	1.16

**Table:** (b) Problem set *large*

*k*	GENIUS-*quality*			GENIUS-*fast*		
	LR	CQ	CER	LR	CQ	CER
2	1.04	0.000	1.04	1.08	0.001	1.08
3	1.04	0.001	1.04	1.12	0.001	1.12
4	1.06	0.002	1.06	1.15	0.002	1.15
5	1.05	0.003	1.05	1.15	0.004	1.15

**Table:** (a) Problem set *middle*

*k*	SOM-*nn*			SOM-*cc*			
	LR	CQ	CER	LR	CQ	CER	TR
2	1.08	0.07	1.01	1.06	0.05	1.01	0.89
3	1.11	0.12	0.98	1.09	0.11	0.98	0.91
4	1.12	0.14	0.95	1.11	0.13	0.96	0.91
5	1.17	0.20	0.94	1.14	0.16	0.94	0.92

**Table:** (b) Problem set *large*

*k*	SOM-*nn*			SOM-*cc*			
	LR	CQ	CER	LR	CQ	CER	TR
2	1.05	0.05	1.00	1.03	0.04	0.99	0.94
3	1.07	0.09	0.96	1.04	0.07	0.97	0.95
4	1.09	0.12	0.94	1.06	0.11	0.94	0.96
5	1.09	0.16	0.91	1.05	0.12	0.91	0.96

**Table 5:** MTSP results, *k* = 3.

Problem	*L*_REF_ [m]	GENIUS-*quality*			GENIUS-*fast*			SOM-*nn*			SOM-*cc*		
		PDM	PDB	*T* [s]	PDM	PDB	*T* [s]	PDM	PDB	*T* [s]	PDM	PDB	*T* [s]
dense_4_	69.3	3.77	0.41	110.3	8.88	2.99	1.6	11.99	1.83	4.3	13.43	0.00	3.8
potholes_2_	58.3	3.03	0.00	185.1	7.44	3.38	1.9	7.23	0.76	6.1	11.58	3.22	5.6
m3_1_	14.8	4.65	0.00	210.5	8.95	2.92	2.3	9.68	0.02	7.3	9.42	0.76	6.4
warehouse_4,*A*_	138.5	5.35	2.13	232.1	9.48	2.80	2.7	10.31	0.00	7.6	8.06	1.23	6.9
warehouse_4,*B*_	153.6	3.10	0.00	271.2	7.57	1.32	3.0	16.58	5.10	7.9	12.65	1.28	6.9
jh_2,*A*_	80.5	5.88	0.00	282.9	11.22	6.12	3.2	18.65	15.86	8.4	14.37	1.54	7.9
jh_2,*B*_	77.8	5.06	1.21	332.6	11.50	5.56	3.0	5.97	0.19	8.7	6.45	0.00	7.7
pb_4_	281.4	4.45	0.00	575.0	10.61	2.58	5.5	10.99	6.50	9.8	10.15	2.57	8.9
ta_2_	129.0	10.22	0.00	1 326.4	20.07	7.36	13.1	8.89	3.33	16.8	6.06	3.29	16.1
h2_5,*A*_	344.3	10.35	1.91	1 635.5	16.70	6.97	18.2	6.43	0.00	58.1	3.81	0.08	52.8
h2_5,*B*_	345.4	10.94	0.00	1 921.4	18.67	9.74	17.5	16.22	11.13	59.1	13.95	5.25	53.1
potholes_1_	97.9	5.18	3.78	5 961.2	10.62	4.85	61.5	6.87	1.22	108.5	6.96	0.00	104.4
jh_1,*A*_	133.0	3.89	0.00	8 397.0	12.77	9.10	86.8	12.78	7.01	184.3	8.93	3.62	172.4
jh_1,*B*_	130.3	4.67	1.95	7 978.1	13.40	7.91	94.5	5.11	0.00	171.9	4.27	0.05	165.5
pb_1.5_	338.7	5.96	0.00	15 727.8	13.45	3.49	224.5	4.98	2.31	163.3	5.95	2.27	154.7
h2_2,*A*_	462.9	10.90	6.57	19 237.8	19.22	11.23	281.8	13.35	2.57	627.4	7.96	0.00	594.0
h2_2,*B*_	478.2	10.18	4.88	24 803.6	16.85	10.33	411.6	4.40	0.91	625.6	3.32	0.00	604.1
ta_1_	202.4	6.00	0.13	32 541.3	14.00	7.31	459.6	16.97	5.05	310.3	6.71	0.00	296.2

**Table 6:** MTSP results, *k* = 5.

Problem	*L*_REF_ [m]	GENIUS-*quality*			GENIUS-*fast*			SOM-*nn*			SOM-*cc*		
		PDM	PDB	*T* [s]	PDM	PDB	*T* [s]	PDM	PDB	*T* [s]	PDM	PDB	*T* [s]
dense_4_	48.8	5.25	0.00	50.4	16.10	5.07	1.2	24.16	13.97	4.0	21.30	11.73	3.6
potholes_2_	38.5	6.55	0.00	126.9	16.58	6.21	1.3	13.39	2.87	5.9	12.25	2.51	5.4
m3_1_	9.9	8.13	0.00	155.9	19.56	10.82	1.7	8.92	2.97	6.5	6.24	2.95	5.8
warehouse_4,*A*_	96.8	4.63	0.08	195.0	13.41	3.53	1.7	11.85	1.51	6.9	8.76	0.00	6.4
warehouse_4,*B*_	123.6	2.92	0.00	188.5	10.01	5.95	1.8	22.85	11.92	7.1	18.03	9.06	6.6
jh_2,*A*_	62.2	3.61	0.00	259.2	11.34	5.48	2.0	17.47	8.82	8.0	17.16	10.82	7.3
jh_2,*B*_	54.6	5.39	0.97	213.8	16.13	6.35	1.9	17.30	0.00	7.9	13.68	1.91	7.2
pb_4_	210.0	11.31	0.00	358.9	23.30	5.85	3.1	23.20	5.73	9.1	12.49	2.16	8.4
ta_2_	105.4	4.91	0.00	806.0	19.10	9.70	6.7	13.50	13.02	15.5	13.63	13.02	14.9
h2_5,*A*_	230.7	8.50	0.00	1 288.8	23.62	9.48	11.5	13.16	7.53	53.9	11.98	5.64	49.0
h2_5,*B*_	253.1	7.93	0.00	1 287.6	18.91	11.22	11.2	21.62	18.68	54.4	18.07	8.82	51.6
potholes_1_	63.9	5.32	1.10	3 574.6	13.36	8.00	35.4	8.03	1.27	100.9	4.60	0.00	98.1
jh_1,*A*_	92.3	4.87	0.00	7 057.5	14.99	9.42	78.9	18.04	7.79	164.2	10.86	2.25	155.6
jh_1,*B*_	86.6	6.50	1.47	7 157.7	16.93	8.96	71.7	13.12	9.08	160.0	7.80	0.00	154.2
pb_1.5_	246.6	8.77	1.23	12 190.4	16.78	6.28	173.1	19.97	6.11	148.0	8.83	0.00	141.9
h2_2,*A*_	315.7	9.99	5.02	15 611.5	21.49	12.20	194.4	1.48	0.00	585.2	0.49	0.12	548.4
h2_2,*B*_	347.3	5.31	0.00	16 162.1	17.70	7.31	289.7	3.83	2.82	575.6	3.66	0.43	551.7
ta_1_	147.8	5.69	0.00	18 867.0	16.11	8.12	286.7	7.90	3.61	282.7	4.85	2.89	269.9

## Symbols

*𝒲* ⊂ *ℝ*^2^: The polygonal domain representing the world to be inspected
*v*: The number of vertices of *𝒲*
*h*: The number of holes of *𝒲*
*v*_*i*_: A vertex of the polygonal domain *𝒲*
**C**: A set of the sensing locations (cities), **C** = {*c*_1_,…, *c*_*n*_}
*n*: The number of cities (sensing locations)
*c*_d_: Starting city (depot), *c*_d_ ∈ **C**
**P**: A set of convex polygons (cells) **P** = {*C*_1_,…, *C*_*r*_}
*r*: The number of convex regions of **P**
*m*: The number of neurons representing a tour
*k*: The number of salesmen
|*s*, *t*|: The Euclidean distance between points *s* and *t*
|*𝒮*(*v*_*i*_, *v*_*j*_)|: The length of the shortest path between two vertices of *𝒲*, *v*_*i*_, *v*_*j*_ ∈ *𝒲*
*ν*_*i*_: A node representing weights of the *i*th neuron
*G*, *μ*, *α*, *d*: Parameters of the used SOM adaptation schema.
